# Associations between Long-Term Air Pollutant Exposures and Blood Pressure in Elderly Residents of Taipei City: A Cross-Sectional Study

**DOI:** 10.1289/ehp.1408771

**Published:** 2015-03-20

**Authors:** Szu-Ying Chen, Chang-Fu Wu, Jui-Huan Lee, Barbara Hoffmann, Annette Peters, Bert Brunekreef, Da-Chen Chu, Chang-Chuan Chan

**Affiliations:** 1 Institute of Occupational Medicine and Industrial Hygiene, College of Public Health, National Taiwan University, Taipei, Taiwan; 2 Division of Surgical Intensive Care, Department of Critical Care Medicine, E-Da Hospital, Kaohsiung, Taiwan; 3 IUF-Leibniz Research Institute for Environmental Medicine and Medical Faculty, Heinrich Heine University of Düsseldorf, Düsseldorf, Germany; 4 Helmholtz Zentrum München–German Research Center for Environmental Health, Neuherberg, Germany; 5 Institute for Risk Assessment Sciences, University of Utrecht, Utrecht, the Netherlands; 6 Institute of Public Health and Community Medicine Research Center, National Yang-Ming University, Taipei, Taiwan

## Abstract

**Background:**

Limited information is available regarding long-term effects of air pollution on blood pressure (BP) and hypertension.

**Objective:**

We studied whether 1-year exposures to particulate matter (PM) and nitrogen oxides (NO_x_) were correlated with BP and hypertension in the elderly.

**Methods:**

We analyzed cross-sectional data from 27,752 Taipei City residents > 65 years of age who participated in a health examination program in 2009. Land-use regression models were used to estimate participants’ 1-year exposures to particulate matter with aerodynamic diameter ≤ 10 μm (PM_10_), coarse particles (PM_2.5–10_), fine particles (≤ 2.5 μm; PM_2.5_), PM_2.5_ absorbance, NO_x_, and nitrogen dioxide (NO_2_). Generalized linear regressions and logistic regressions were used to examine the association between air pollution and BP and hypertension, respectively.

**Results:**

Diastolic BP was associated with 1-year exposures to air pollution, with estimates of 0.73 [95% confidence interval (CI): 0.44, 1.03], 0.46 (95% CI: 0.30, 0.63), 0.62 (95% CI: 0.24, 0.99), 0.34 (95% CI: 0.19, 0.50), and 0.65 (95% CI: 0.44, 0.85) mmHg for PM_10_ (10 μg/m^3^), PM_2.5–10_ (5 μg/m^3^), PM_2.5_ absorbance (10^–5^/m), NO_x_ (20 μg/m^3^), and NO_2_ (10 μg/m^3^), respectively. PM_2.5_ was not associated with diastolic BP, and none of the air pollutants was associated with systolic BP. Associations of diastolic BP with PM_10_ and PM_2.5_ absorbance were stronger among participants with hypertension, diabetes, or a body mass index ≥ 25 kg/m^2^ than among participants without these conditions. One-year air pollution exposures were not associated with hypertension.

**Conclusions:**

One-year exposures to PM_10_, PM_2.5–10_, PM_2.5_ absorbance, and NO_x_ were associated with higher diastolic BP in elderly residents of Taipei.

**Citation:**

Chen SY, Wu CF, Lee JH, Hoffmann B, Peters A, Brunekreef B, Chu DC, Chan CC. 2015. Associations between long-term air pollutant exposures and blood pressure in elderly residents of Taipei City: a cross-sectional study. Environ Health Perspect 123:779–784; http://dx.doi.org/10.1289/ehp.1408771

## Introduction

The 2010 American Heart Association (AHA) scientific statement on particulate air pollution asserts that increased arterial blood pressure (BP) is an important outcome of acute particulate matter (PM) exposures and a possible biological mechanism linking PM exposure to cardiovascular disease (Brook et al. 2010). However, less is known about the role of long-term PM exposures on BP. Several studies have investigated the impacts of long-term PM exposures with respect to BP (Auchincloss et al. 2008; Chuang et al. 2011; Dong et al. 2013; Fuks et al. 2011, 2014; Liu et al. 2014; Schwartz et al. 2012; Sørensen et al. 2012); however, the results remain inconclusive. An epidemiological study first reported that average 30-day exposures to fine particles (≤ 2.5 μm; PM_2.5_) were positively associated with pulse pressure and systolic BP (Auchincloss et al. 2008). Epidemiological studies in Europe and the United States further reported that average 1-year exposures to PM_2.5_ or black carbon were positively associated with both systolic and diastolic BP (Fuks et al. 2011; Schwartz et al. 2012). Additionally, two cross-sectional studies in Asian countries found positive associations between long-term exposure to PM and increased BP and hypertension (Chuang et al. 2011; Dong et al. 2013). However, reported associations between air pollution and BP have differed among studies, possibly because of differences in characteristics among study cohorts. For example, a Danish cohort found that 1- and 5-year exposures to nitrogen oxides (NO_x_), a traffic-emitted indicator, were associated with a decrease in BP among middle-aged subjects (Sørensen et al. 2012), whereas Liu et al. (2014) reported no associations between long-term traffic-related air pollution exposures and BP in children 10 years of age. The largest multicenter study to date by Fuks et al. (2014) used 15 population-based cohorts, participating in the European Study of Cohorts for Air Pollution Effects (ESCAPE), to investigate the effects of residential exposures to PM and nitrogen oxides on BP and prevalent hypertension in European populations. They reported that systolic and diastolic BP in non-antihypertensive medicated participants were associated with traffic load on major roads within 100 m of residence, but not with concentrations of air pollutants modeled by land use regression (LUR). The odds ratio for prevalent hypertension was also elevated with traffic load (Fuks et al. 2014).

The inconsistent findings of associations between long-term exposures to air pollution and BP may be attributed to the heterogeneity of PM sizes or compositions between study locations. Some studies have shown that BP was positively associated with PM in the presence of roadway traffic or traffic-related particles (Auchincloss et al. 2008; Fuks et al. 2014; Schwartz et al. 2012). Differences in exposure measurements also may contribute to diverse findings on associations between PM and BP among studies. The use of fixed-site monitoring data to assess residential PM exposures, as described in the studies by Auchincloss et al. (2008), Chuang et al. (2011), and Dong et al. (2013), most likely result in the misclassification of exposures that can eventually bias the research findings. LUR models have been successfully applied in the ESCAPE project to model residential long-term air pollution on a small spatial scale to a spatial resolution of 25 m, reflecting within-city differences in traffic-related emissions among European cities (Eeftens et al. 2012; Wang et al. 2013). In this study, we applied the LUR model based on the ESCAPE protocol to estimate 1-year exposures to different sizes and compositions of air pollution, including particulate matter with aerodynamic diameter ≤ 10 μm (PM_10_), coarse particles (PM_2.5–10_), PM_2.5_, PM_2.5_ absorbance, NO_x_, and nitrogen dioxide (NO_2_), and investigated whether BP and hypertension were associated with these air pollutants in elderly participants living in Taipei City.

## Methods

*Study population.* We used a cross-sectional design in this study and the study population was selected from the Taipei City Elderly Health Screening Program in 2009 [http://health.gov.taipei/Default.aspx?tabid=401 (in Chinese)]. This program is an annual program run by the Department of Health of Taipei City Government from 1 March to 31 August. All senior citizens > 65 years old and residing in Taipei City are invited to participate in this health screening program every 3 years. The Department of Health of Taipei City Government prepared a data set, which has been decoded and has delinked personal information of names and identification from medical records, for medical research. We were allowed to use these data for our study after our application for use was approved by Department of Health of Taipei City Government. Overall, 42,105 participants participated in this program in 2009. After we excluded 11,666 participants with missing information on variables used in the analysis or used to estimate exposure, 30,439 participants were candidates for this study. Using the validation data from electronic sphygmomanometers (Christofaro et al. 2009), subjects whose measured BP values were > 190 mmHg or < 90 mmHg for systolic BP and > 120 mmHg or < 60 mmHg for diastolic BP were further excluded to minimize the potential for measurement errors in BP values. This gave us a final total of 27,752 subjects recruited for this study. This study was approved by the Joint Institutional Review Board of the Public Health College of the National Taiwan University.

*Health assessment.* The health screening program was conducted in 10 branches of the Taipei Municipal Hospital and consisted of a clinician interview, self-reported questionnaire, and venous biochemistry sampling. The clinician interview record and questionnaire provided information including home address, age, sex, height, weight, body mass index (BMI), education level, smoking status, alcohol consumption, betel nut–chewing status, and physical activity, as well as a history of physician-diagnosed hypertension and diabetes for each subject.

The study subjects’ BP measurements, that is, systolic and diastolic BP, were taken by trained medical personnel using electronic sphygmomanometers (model HEM-770A; Omron Health Care). All BP measurements were performed in the morning (0800 to 1000 hours). After completion of the 30-min questionnaire interview, BP was measured once in a seated, upright position. One of three cuff sizes (adult standard, large, and thigh-sized) was used depending on the circumference of the subject’s left upper arm. To minimize the potential effects of antihypertensive drugs, participants were instructed to not take any medication the morning of the BP measurement. In this study, subjects who either had self-reported physician-diagnosed hypertension (subjects had been diagnosed with hypertension and used antihypertensive medication) or had measured unknown hypertension (measured BP values ≥ 140 mmHg for systolic BP or ≥ 90 mmHg for diastolic BP but had never been diagnosed with hypertension) were all defined as hypertensive subjects.

*Exposure assessment.* We used the LUR to model the annual average concentrations of different sizes and compositions of air pollution, including PM_10_, PM_2.5–10_, PM_2.5_, PM_2.5_ absorbance, NO_x_, and NO_2_, for each participant. This modeling approach was derived and developed from the ESCAPE project (http://www.escapeproject.eu/manuals) (Eeftens et al. 2012; Wang et al. 2013). In summary, NO_x_ and NO_2_ were measured for three 14-day periods during intermediate season (October–December 2009), cold season (January–March 2010), and warm season (June–August 2010) at 40 spatially distributed sampling sites in Taipei. The average temperature and relative humidity were 21.0°C and 76%, 18.2°C and 78%, and 28.8°C and 74% for the three seasons, respectively. Ogawa passive badges (Ogawa USA Inc.) were used to measure NO_x_ and NO_2_ concentrations at urban backgrounds and streets. PM_2.5_ and PM_10_ were measured at 20 of the 40 sampling sites using Harvard impactors (Air Diagnostics and Engineering Inc.). The collected filters were then measured using Smoke Stain Reflectometer (model 43; Diffusion Systems Ltd.) to determine PM_2.5_ absorbance. Land use data and traffic-related information were combined with measured concentrations to derive LUR models using supervised forward stepwise multiple regressions. For traffic variables, circular buffers with radii of 25, 50, 100, 300, 500, and 1,000 m around each site were calculated. For land use and population, buffers of 100, 300, 500, 1,000, and 5,000 m were calculated. Different road types, including major roads (national highway, provincial highway, expressway, and city street), elevated highway (roads established above ground level or highway ramp), as well as all roads were considered in LUR models. The fit of the final LUR models for each of the traffic-related air pollutants was good, with cross-validated *R*^2^ values of 0.74, 0.52, 0.91, 0.92, 0.75, and 0.63 for PM_10_, PM_2.5–10_, PM_2.5_, PM_2.5_ absorbance, NO_x_, and NO_2_, respectively. Exposure modeling for NO_x_ and NO_2_ followed the procedure outlined by Lee et al. (2014), and the statistics for land use variables are summarized in Supplemental Material, Table S1. Each participant’s annual average exposures to the six air pollutants were calculated by inserting the values of the land use variables at their residential addresses into our LUR models.

*Statistical analyses.* ArcGIS (ESRI) was used to obtain geographic information system (GIS) information used in the air pollution exposure modeling. Generalized linear regression models were applied to examine associations between 1-year exposures to air pollutants and BP in elderly persons > 65 years of age. We applied the variable selection technique, 10% change-in-estimate criterion (Rothman et al. 2008), to select potential confounding factors. In addition, the mean-centered square terms of continuous variables were included in the modeling if nonlinearity was present, checked by a scatter plot between the response variable and the predictor. The covariates of sex, age, age mean-centered square, BMI (body weight divided by the square of height measured at the health examination), BMI mean-centered square, smoking status (current smoker or former smoker), alcohol consumption (< 1 drink/week, 1–3 drinks/week, or > 3 drinks/week), education (primary school or less, up to secondary school or equivalent, or university degree or more), hypertension (classified as physician-diagnosed hypertension or measured unknown hypertension), and diabetes (subjects had been diagnosed with diabetes and use of antidiabetic medication or fasting glucose > 126 mg/dL in the health examination), were finally identified as adjustment variables in the main model. In the extended model, we further adjusted for traffic proximity (distance to the nearest major road) in addition to the selected individual covariates in the main model to consider the possible effect of traffic noise. To account for the influence of individual comorbidities, analyses were performed to examine whether the association between BP and air pollution was modified by hypertension, diabetes, or obesity (BMI ≥ 25 kg/m^2^). We also estimated associations stratified according to a history of physician-diagnosed hypertension versus no previous diagnosis of hypertension in addition to stratifying on hypertension (including measured but not diagnosed) versus no hypertension. We examined the effect modification in the regression models by including interaction terms between air pollution and individual comorbidity category.

We used logistic regression models to estimate associations between 1-year average exposures to air pollutants and prevalent hypertension, with participants with a previous diagnosis and those with elevated systolic or diastolic BP at the time of the study examination classified as cases. In addition, we estimated associations with prevalent hypertension based on a previous physician diagnosis only, after excluding participants with elevated BP at the study examination only. To compare with other ESCAPE cohorts, all of the estimated effects were uniformly presented as the mean and 95% confidence interval (CI) for BP values in increments of 10 μg/m^3^ for PM_10_, 5 μg/m^3^ for PM_2.5–10_ and PM_2.5_, 10^–5^/m for PM_2.5_ absorbance, 20 μg/m^3^ for NO_x_, and 10 μg/m^3^ for NO_2_. We also estimated associations with interquartile range (IQR) increases in the pollutants. An alpha level of 0.1, with a two-tailed distribution, was used to determine statistical significance for effect modification. All of the analyses were performed using SAS software (version 9.1.3; SAS Institute Inc.).

## Results

The basic characteristics of the 27,752 study subjects are summarized in Table 1. Our study population consisted of senior retired residents with a mean age of 74.8 years and a nearly equal sex distribution. Of all study subjects, 30.9% had a BMI > 25 kg/m^2^. Among 17,428 hypertensive subjects, 12,702 were self-reported physician-diagnosed hypertension and 4,726 belonged to measured unknown hypertension.

**Table 1:** Basic characteristics of study population (*n *= 27,752).

Variable	Mean ± SD or *n* (%)
Age (years)	74.8 ± 6.4
Body mass index (kg/m^2^)	24.3 ± 3.4
Systolic blood pressure (mmHg)	136.3 ± 17.6
Diastolic blood pressure (mmHg)	76.8 ± 10.2
Male sex	14,414 (51.9)
Education level
Low (primary school or less)	9,585 (34.5)
Medium (up to secondary school or equivalent)	10,103 (36.4)
High (university degree and more)	8,064 (29.1)
Alcohol consumption (drinks/week)
< 1	22,205 (80.0)
1–3	4,972 (17.9)
> 3	575 (2.1)
Smoking
Current smoker	1,868 (6.7)
Former smoker	25,584 (93.3)
Physical activity (hr/week)
< 1	16,795 (60.5)
1–3	8,160 (29.4)
> 3	2,792 (10.1)
Hypertension	17,428 (62.8)
Physician-diagnosed hypertension^*a*^	12,702 (45.8)
Measured unknown hypertension^*b*^	4,726 (17.0)
Diabetes^*c*^	3,557 (12.8)
^***a***^Subjects had been diagnosed with hypertension and used antihypertensive medication. ^***b***^Subjects with measured BP values ≥ 140 mmHg for systolic BP or ≥ 90 mmHg for diastolic BP but had never been diagnosed with hypertension. ^***c***^Physician-diagnosed diabetes and use of antidiabetic medication or fasting glucose > 126 mg/dL in the health examination.	

Table 2 shows the 1-year average concentrations of six air pollutants for the 27,752 subjects. The annual average concentrations of NO_2_ and PM_10_ were 23.7 μg/m^3^ and 47.3 μg/m^3^, respectively, which were below the National Ambient Air Quality Standards of the Taiwan Environmental Protection Agency (50 ppb for NO_2_ and 65 μg/m^3^ for PM_10_) [http://ivy5.epa.gov.tw/epalaw/docfile/040060.pdf (in Chinese)]; however, the 1-year average concentration of PM_2.5_ (24.5 μg/m^3^) exceeded the National Ambient Air Quality Standards of Taiwan (15 μg/m^3^ for PM_2.5_). PM_10_ was moderately correlated with PM_2.5–10_ (*r* = 0.67), PM_2.5_ (*r* = 0.40), PM_2.5_ absorbance (*r* = 0.62), NO_x_ (*r* = 0.53), and NO_2_ (*r* = 0.53) (see Supplemental Material, Table S2). PM_10_, PM_2.5_ absorbance, NO_x_, and NO_2_ were moderately correlated with the lengths of all major roads in 25, 50, 100, and 500 m buffer zones (*r* = 0.35–0.61).

**Table 2:** One-year average concentrations of six air pollutants for 27,752 subjects.

Exposures	Mean ± SD	IQR	Range
PM_10_ (μg/m^3^)	47.3 ± 4.0	5.3	(36.1–63.9)
PM_2.5–10_ (μg/m^3^)	21.2 ± 3.5	5.2	(15.9–33.9)
PM_2.5_ (μg/m^3^)	24.5 ± 3.9	4.0	(12.8–48.2)
PM_2.5_ absorbance (10^–5^/m)	1.8 ± 0.3	0.4	(1.1–3.1)
NO_x_ (μg/m^3^)	38.4 ± 15.3	17.5	(0.1–78.7)
NO_2_ (μg/m^3^)	23.7 ± 5.8	6.7	(4.2–37.2)

We estimated statistically significant (*p* < 0.05) positive associations of average 1-year exposures to PM_10_, PM_2.5–10_, PM_2.5_ absorbance, NO_x_, and NO_2_ with diastolic BP, which were consistent among the different model specifications (Table 3). In the crude model, 1-year exposures to PM_10_, PM_2.5–10_, PM_2.5_ absorbance, NO_x_, and NO_2_ were associated with higher diastolic BP. After adjusting for individual covariates (main model), diastolic BP was associated with 1-year exposures to air pollution, with estimates of 0.73 (95% CI: 0.44, 1.03), 0.46 (95% CI: 0.30, 0.63), 0.62 (95% CI: 0.24, 0.99), 0.34 (95% CI: 0.19, 0.50), and 0.65 (95% CI: 0.44, 0.85) mmHg with increments of 10 μg/m^3^ for PM_10_, 5 μg/m^3^ for PM_2.5–10_, 10^–5^/m for PM_2.5_ absorbance, 20 μg/m^3^ for NO_x_, and 10 μg/m^3^ for NO_2_, respectively. The associations between diastolic BP and an IQR increment of PM_10_, PM_2.5–10_, PM_2.5_ absorbance, NO_x_, and NO_2_, was 0.29–0.49 mmHg in the extended model (see Supplemental Material, Table S3). Diastolic BP also was positively associated with air pollutants (other than PM_2.5_) after further adjustment for proximity to traffic (Table 3, extended model). Diastolic BP was not associated with PM_2.5_ mass concentration in crude or adjusted models. In addition, none of the air pollutants was significantly associated with systolic BP (Table 3; see also Supplemental Material, Table S3).

**Table 3:** Associations of systolic and diastolic blood pressures with annual averages of particulate matter and nitrogen oxides.

Exposures (increment)/models	mmHg (95% CI)	
	Systolic BP	Diastolic BP
PM_10_ (10 μg/m^3^)
Crude	0.21 (–0.31, 0.73)	0.75 (0.45, 1.05)
Main	0.33 (–0.17, 0.84)	0.73 (0.44, 1.03)
Extended	0.45 (–0.08, 0.99)	0.77 (0.46, 1.09)
PM_2.5–10_ (5 μg/m^3^)
Crude	–0.11 (–0.40, 0.19)	0.50 (0.33, 0.67)
Main	–0.03 (–0.31, 0.26)	0.46 (0.30, 0.63)
Extended	–0.01 (–0.30, 0.28)	0.46 (0.29, 0.63)
PM_2.5_ (5 μg/m^3^)
Crude	–0.01 (–0.28, 0.25)	–0.09 (–0.24, 0.06)
Main	0.08 (–0.17, 0.34)	–0.02 (–0.18, 0.13)
Extended	0.11 (–0.15, 0.38)	–0.05 (–0.20, 0.11)
PM_2.5_ absorbance (10^–5^/m)
Crude	–0.06 (–0.73, 0.60)	0.72 (0.34, 1.10)
Main	0.15 (–0.49, 0.78)	0.62 (0.24, 0.99)
Extended	0.26 (–0.41, 0.94)	0.63 (0.23, 1.03)
NO_x_ (20 μg/m^3^)
Crude	–0.16 (–0.43, 0.11)	0.33 (0.17, 0.49)
Main	–0.00 (–0.26, 0.26)	0.34 (0.19, 0.50)
Extended	0.07 (–0.23, 0.37)	0.41 (0.23, 0.59)
NO_2_ (10 μg/m^3^)
Crude	–0.04 (–0.40, 0.31)	0.65 (0.45, 0.86)
Main	0.17 (–0.18, 0.51)	0.65 (0.44, 0.85)
Extended	0.28 (–0.10, 0.66)	0.74 (0.52, 0.97)
The main models were calculated by generalized linear models, adjusted for sex, age, age mean-centered square, BMI, BMI mean-centered square, smoking status, alcohol consumption, education, hypertension, and diabetes. The extended models were further adjusted for traffic proximity in addition to covariates in the main models.		

**Figure 1:**
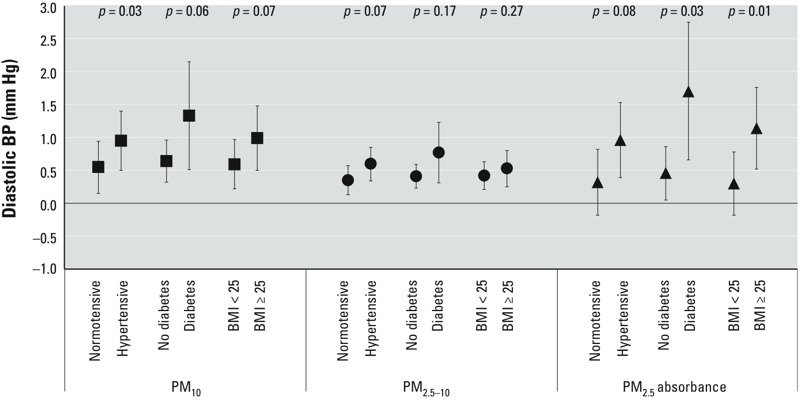
Estimates (95% CIs) of diastolic blood pressure in association with an increment of 10 μg/m^3^ for PM_10_, 5 μg/m^3^ for PM_2.5–10_, and 10^–5^/m for PM_2.5_ absorbance stratified by hypertensive (physician-diagnosed hypertension or measured unknown hypertension), diabetic (physician-diagnosed diabetes and use of antidiabetic medication or fasting glucose > 126 mg/dL in health examination), or obese (BMI ≥ 25 kg/m^2^) status. The estimates were calculated by generalized linear models, adjusted for sex, age, age mean-centered square, smoking status, alcohol consumption, education, traffic proximity, and individual comorbid conditions other than analyzed stratum.

Figure 1 illustrates the associations between diastolic BP and PM_10_, PM_2.5–10_, and PM_2.5_ absorbance stratified by an individual’s comorbidities, including hypertension, diabetes, or obesity. We found 0.95 mmHg (95% CI: 0.50, 1.40), 1.33 mmHg (95% CI: 0.51, 2.15), and 0.99 mmHg (95% CI: 0.50, 1.48) increase in diastolic BP for an increment of 10 μg/m^3^ for PM_10_ in subjects with hypertension, diabetes, or a BMI ≥ 25 kg/m^2^, respectively, which were higher than the values for subjects without hypertension (0.55 mmHg; 95% CI: 0.15, 0.94), diabetes (0.64 mmHg; 95% CI: 0.32, 0.96), or with a BMI < 25 kg/m^2^ (0.59 mmHg; 95% CI: 0.22, 0.97). The *p*-values for interaction of hypertension, diabetes, and obesity with PM_10_ in the model were 0.03, 0.06, and 0.07, respectively. Similar results of stratified analyses results were observed for the association of PM_2.5_ absorbance with diastolic BP. The *p*-values for interaction of the three comorbidity categories with PM_2.5_ absorbance were 0.08, 0.03, and 0.01, respectively. The increase in diastolic BP with exposure of PM_2.5–10_ was also higher in subjects with hypertension than without hypertension (*p*-value of interaction: 0.07), but associations were only slightly stronger among those with diabetes or obesity (interaction *p*-values = 0.17 and 0.27, respectively). The associations between diastolic BP and NO_x_ or NO_2_ did not differ according to comorbidity categories (data not shown). Estimates of associations with systolic or diastolic BP stratified according to a previous diagnosis of hypertension (*n* = 12,702) versus no previous history of hypertension (*n* = 15,050, including participants with elevated systolic or diastolic BP at the study examination) (see Supplemental Material, Table S4), were generally consistent with stratum-specific estimates when hypertension also included participants with elevated SBP or DBP at the study examination but no history of physician-diagnosed hypertension (as shown for selected exposure and diastolic BP in Figure 1).

Table 4 shows the odds ratios of hypertension prevalence with 1-year exposures to PM and nitrogen oxides. For both the main and extended models, we did not find any associations between prevalence of hypertension and 1-year exposures to air pollution in the 27,752 subjects enrolled in the study. Still, no associations were found between hypertension and air pollution in the subset of 23,026 participants from which subjects with measured unknown hypertension were excluded to consider the misclassification of hypertension by BP measurement error.

**Table 4:** Estimated odds ratios (95% CIs) for the prevalence of hypertension with 1-year exposures to particulate matter and nitrogen oxides.

Exposures (increment)/models	All subjects (*n *= 27,752)	Measured unknown hypertension excluded (*n *= 23,026)
PM_10_ (10 μg/m^3^)
Main	1.000 (0.939, 1.066)	0.983 (0.917, 1.054)
Extended	1.001 (0.936, 1.071)	0.976 (0.907, 1.051)
PM_2.5–10_ (5 μg/m^3^)
Main	0.996 (0.961, 1.033)	1.003 (0.964, 1.043)
Extended	0.997 (0.961, 1.033)	1.002 (0.963, 1.042)
PM_2.5_ (5 μg/m^3^)
Main	0.991 (0.959, 1.023)	0.976 (0.942, 1.011)
Extended	0.991 (0.958, 1.024)	0.972 (0.937, 1.009)
PM_2.5_ absorbance (10^–5^/m)
Main	0.926 (0.854, 1.003)	0.921 (0.842, 1.001)
Extended	0.918 (0.843, 1.001)	0.917 (0.835, 1.001)
NO_x_ (20 μg/m^3^)
Main	0.990 (0.958, 1.023)	0.978 (0.944, 1.014)
Extended	0.988 (0.951, 1.026)	0.967 (0.927, 1.009)
NO_2_ (10 μg/m^3^)
Main	0.999 (0.956, 1.043)	0.987 (0.941, 1.034)
Extended	0.999 (0.952, 1.048)	0.979 (0.929, 1.032)
The main models were calculated by logistic regression models, adjusted for sex, age, age mean-centered square, BMI, BMI mean-centered square, smoking status, alcohol consumption, education, and diabetes. The extended models were further adjusted for traffic proximity in addition to individual covariates in the main models.		

## Discussion

Our study results demonstrate a positive association between diastolic BP and 1-year exposures to air pollution among people > 65 years of age by use of the large study population and the comprehensive residential address information to improve the estimation of annual average air pollution exposures. In addition, associations of PM_10_, PM_2.5–10_, and PM_2.5_ absorbance were stronger among those with hypertension, diabetes, or obesity than among participants without these comorbid conditions. Although the point estimates were small and bias cannot be ruled out, they add to existing evidence suggesting that air pollution exposures may have a substantial public health impact, especially in vulnerable populations, given the ubiquitous nature of these exposures.

The annual average concentrations of PM_10_ and PM_2.5_ for participants in this study were fairly high compared with those in other ESCAPE cohorts (Fuks et al. 2014). The positive findings in associations between different sizes in PM and diastolic BP may be attributable to high particulate concentrations in Taipei. Vehicular emissions and road dust are principal components of PM_10_ and PM_2.5–10_ in the Taipei metropolitan area (Liang et al. 2013). Reflectance measurements of PM_2.5_ absorbance have been shown to correlate well with actual measurements of elemental carbon and can be considered a marker for traffic emissions (diesel soot). The exposure data in this study showed that PM_2.5_ absorbance was correlated with the lengths of all major roads in 25-, 50-, 100-, and 500-m buffer zones. This finding suggests that associations between diastolic BP and PM_2.5_ absorbance may have been driven by effects of traffic-emitted PM_2.5_ components. Hoffmann et al. (2009) found an association between residential traffic road proximity and decrease in ankle-brachial index. Schwartz et al. (2012) found significant positive associations between systolic BP, as well as diastolic BP and 1-year averaged black carbon levels. These findings also support that traffic-emitted particles are key components relevant to cardiovascular health.

The role of traffic-related pollution is further supported by the fact that we observed that two other air pollutants, NO_x_ and NO_2_, were also positively associated with diastolic BP. Previously, limited information was available on associations of NO_x_ and NO_2_ with BP, and the results remain inconclusive. Bilenko et al. (2015) recently reported a positive association between long-term NO_2_ and diastolic BP in children. Two other studies reported weak inverse associations between long-term exposures to NO_x_ and NO_2_ and BP (Liu et al. 2014; Sørensen et al. 2012), whereas subgroup analyses by Sørensen et al. (2012) found that 5 years of exposure to NO_x_ was weakly but not significantly associated with systolic BP in subjects with a past history of cardiovascular disease. In the ESCAPE analysis of > 10,000 participants, NO_2_ showed a weak inverse relationship with systolic BP in nonmedicated participants, but not in medicated participants (Fuks et al. 2014). More studies are therefore necessary to understand the relationships between NO_x_ and NO_2_ and BP.

Furthermore, the stratified analyses found stronger associations between 1-year PM_10_ and PM_2.5_ absorbance exposures and BP among subjects with hypertension, diabetes, or obesity, which are three important determinants of metabolic syndrome. But we did not find any associations between PM_2.5_ or any of the other exposures and systolic BP. Auchincloss et al. (2008) found that 30-day exposure to PM_2.5_ was associated with higher systolic BP and pulse pressure in subjects with hypertension or antihypertensive medication prescription. Some possible biomechanisms, including systemic inflammation, oxidative stress, and endothelial dysfunction, may contribute to the enhanced association between BP and long-term exposures to PM in these vulnerable subjects (Brook and Rajagopalan 2009; Dubowsky et al. 2006). Our findings suggest that these people are more vulnerable to long-term air pollution effects and should be well educated about the potential impacts of long-term air pollution exposures.

Previous studies have reported a stronger association between BP and air pollution among subjects with antihypertensive medication (Auchincloss et al. 2008; Fuks et al. 2014; Schwartz et al. 2012). Although the information on antihypertensive medication was not available in our study, we believed that the study subjects who had a history of physician-diagnosed hypertension should have been prescribed with antihypertensive medication, which has been mandatory for reimbursement under the implementation of National Health Insurance in Taiwan since 1995. Therefore, subgroup analyses stratified by self-reported physician-diagnosed hypertension may reflect the effect of antihypertensive medication on the BP–air pollution relationship in our study (see Supplemental Material, Table S4). We found that estimates for diastolic BP with exposures to PM_10_, PM_2.5–10_, and PM_2.5_ absorbance were stronger in subjects with a history of physician-diagnosed hypertension in comparison with subjects who denied a history of physician-diagnosed hypertension. Such results were different from the findings by Fuks et al. (2014), who reported an increase in BP in nonmedicated participants. Our findings imply that the BP–air pollution relationship may be modified primarily by study subject’s hypertensive status. Also, a rebound in BP may contribute to a higher diastolic BP response to air pollution among subjects with physician-diagnosed hypertension because participants were asked in advance not to take their antihypertensive medication in the morning when they came to do their physical examination.

In this study, we observed isolated elevations in diastolic BP, but not systolic BP, with 1-year exposures to air pollution. Systolic BP is considered a major cardiovascular disease risk factor, especially in elderly. In general, there is a linear rise in systolic BP after 30 years of age, but diastolic BP declines with age after 50–60 years (Franklin et al. 1997). The negative finding of the association between systolic BP and 1-year air pollution exposures may be attributable to a much larger effect of traditional risk factor, for example, age, on systolic BP than 1-year air pollution exposures among our study subjects. Diastolic BP is, to some extent, associated with the trend of arterial resistance. The increase in diastolic BP may reflect the loss of elasticity as well as the progressive diffusion of atherosclerotic lesions with exposures to air pollution. Previous studies also suggested that isolated diastolic hypertension is associated with increased risk of specific cardiovascular disease, such as stroke, aortic aneurysm, or peripheral occlusive artery disease (Arima et al. 2011; Rapsomaniki et al. 2014). In addition, diastolic BP, a relatively stable component of the arterial sphygmogram, may be more likely to be associated with air pollution by one single BP measurement in this study. By contrast, systolic BP, which is considered the dynamic component of BP and more closely linked to variations in pulse pressure, is more easily influenced by study subject’s short-term activity before measurements. A single measurement of systolic BP may result in wider variation than diastolic BP and bias the association between air pollution and systolic BP toward the null in this study.

Average 1-year exposures to air pollution were not associated with the prevalence of hypertension in the present study. The definition of hypertension in this study was based not only on self-reported physician-diagnosed hypertension but also on measured unknown hypertension; however, the single measurement of BP values may have led to a false positive assignment of hypertension. Such misclassification may have biased the results. Still the associations between hypertension and 1-year exposures to air pollutants were not found when subjects with measured unknown hypertension were excluded. Another possible explanation for this null result is that the study population volunteered to participate in the health screening program and might be healthier than other elders. Our study population had a comparatively higher educational attainment level and lower prevalence of health-risk behaviors, that is, alcohol consumption and smoking, than the age-matched population in Taiwan (Liang et al. 2004). This “healthy survivor effect” may bias the effect estimates towards to the null.

Several limitations of our study should be noted. We did not control for the individual or neighborhood levels of socioeconomic status. In general, education, occupation, and income are considered important determinants for socioeconomic status. We controlled for education attainment, but not occupation and income because relevant data were lacking. However, the effect of occupation on health behaviors could be ignored because our study subjects were all > 65 years, the mandatory retirement age in Taiwan. On the other hand, some studies found insignificant effects on individuals’ health by income equivalent scales in Taiwan since the implementation of National Health Insurance in 1995, especially in the elderly (Hsiao and Cheng 2013; Leon-Gonzalez and Tseng 2011). Therefore, we believe that educational attainment is the best available indicator of socioeconomic status in our study. Another possible unmeasured confounder is traffic noise, which has been demonstrated to be associated with increased BP and other cardiovascular outcomes (Babisch 2006; Chang et al. 2007). However, associations between diastolic BP and air pollutants did not change substantially with adjustment for traffic proximity, an important predictor for traffic noise modeling (Chang et al. 2012). Additionally, neither time-activity of the participants nor vertical distance of residency were considered in this study. The information on time spent indoors or type of residential ventilation was not available in this study. Air pollution exposures for subjects who lived in mid- to high-level buildings (fourth floor or higher) can be overestimated up to two times for PM and one and a half times for nitrogen oxides (Chu et al. 2011; Wu et al. 2014) by our LUR models, which may result in exposure misclassification. Last, because the study subjects were all > 65 years and from a single geographical area, additional investigations are needed to confirm whether the study results can be extrapolated to younger populations and people in different geographical areas.

Regardless of these limitations, average 1-year exposures to PM (other than PM_2.5_) and nitrogen oxides were associated with higher diastolic BP among Taipei City residents > 65 years of age. In addition, associations of diastolic BP with PM_10_ and PM_2.5_ absorbance were stronger among those with hypertension, diabetes, or obesity than among participants without these comorbid conditions. Further cohort studies with repeated measures are necessary to confirm the causal relationship between long-term air pollution and changes in blood pressure.

## Supplemental Material

(224 KB) PDF
