# Supplementary material for: Associations between Long-Term Air Pollutant Exposures and Blood Pressure in Elderly Residents of Taipei City: A Cross-Sectional Study
Source: Environ Health Perspect. 2015 Mar 20;123(8):779–84. doi: 10.1289/ehp.1408771 (PMC4529013; doi:10.1289/ehp.1408771)
Supplement: (224 KB) PDF [file ehp.1408771.s001.acco.pdf]

Environ Health Perspect
doi: 10.1289/ehp.1408771

Note to Readers: ***EHP*** **strives to ensure that all journal content is accessible to all readers. However, some figures and Supplemental Material published in *EHP* articles may not conform to 508 standards due to the complexity of the information being presented. If you need assistance accessing journal content, please contact ehp508@niehs.nih.gov. Our staff will work with you to assess and meet your accessibility needs within 3 working days.**

Supplemental Material

Associations between Long-Term Air Pollutant Exposures and Blood Pressure in Elderly Residents of Taipei City: A Cross-Sectional Study

Szu-Ying Chen, Chang-Fu Wu, Jui-Huan Lee, Barbara Hoffmann, Annette Peters, Bert Brunekreef, Da-Chen Chu, and Chang-Chuan Chan

Table of Contents

Table S1. Summary of the statistics for land use variables of the exposure modelling for six air pollutants.

Table S2. Spearman correlation coefficients for air pollutants and the total lengths of major roads in 25, 50, 100, and 500 buffer zones.

Table S3. Associations of systolic and diastolic blood pressures with an interquartile range increment of particulate matter and nitrogen oxides.

Table S4. Subgroup analyses for associations of systolic and diastolic blood pressures with increments of annual averages of particulate matter and nitrogen oxides stratified by physician-diagnosed hypertension.

Table S1. Summary of the statistics for land use variables of the exposure modelling for six air pollutants.

Air pollution	Land use equation	R^2 of model	R^2 validation	RMSE (validation)	Number of sites	Measured concentration
PM_{10}	34.41 + 3.41 × 10^{-2} × road area, 50 m + 1.10 × 10^{-4} × industrial area, 5000 m + 2.46 × 10^{-5} × commercial area, 1000 m + 1.16 × 10^{-3} × construction area 100 m	0.87	0.74	3.05 μg/m^3	20	48.6 ± 5.9 [39.2 - 64.0] μg/m^3
$PM_{2.5-10}$	13.88 + 1.20 × 10^{-6} × industrial area, 5000 m + 2.23 × 10^4 × inverse distance squared to the nearest elevated road + 2.97 × 10^{-5} × commercial area, 1000 m	0.65	0.52	3.15 μg/m^3	20	23.3 ± 3.1 [18.6 - 31.3] μg/m^3
$PM_{2.5}$	13.81 + 2.23 × 10^{-3} × road area, 50 m + 72.07 × inverse distance to the nearest major road + 9.89 × 10^{-5} × industrial are, 500 m + 2.22 × 10^{-3} × construction area, 100 m + 1.13 × 10^{-5} × residential area, 1000 m - 5.62 × 10^{-7} × river area, 5000 m	0.95	0.91	1.75 μg/m^3	20	26.0 ± 5.6 [17.4 - 40.6] μg/m^3
$PM_{2.5}$ absorbance	1.07 + 5.76 × 10^{-6} × transport building area, 100 – 500 m + 8.69 × 10^{-4} × road area, 25 m + 3.75 × 10^{-5} × transport building area, 100 m + 4.40 × 10^{-6} × industrial area, 500 m + 2.17 × 10^{-5} × length elevated road, 1000 m + 6.93 × 10^{-5} × commercial area, 100 m	0.96	0.92	0.11 10^{-5} m^{-1}	20	2.0 ± 0.4 [1.2 - 2.6] 10^{-5} m^{-1}
NO_x	47.57 + 0.28 × length major road, 25 m - 2.91 × 10^{-4} × urban green area, 300 m - 3.43 × 10^{-6} × urban green area, 300 – 5000 m + 1.24 × 10^{-3} × length major road, 50 – 500 m + 5.80 × 10^{-2} × length major road, 25 – 50 m - 1.32 × 10^{-4} × natural area, 500 m	0.81	0.75	10.70 μg/m^3	40	72.4 ± 22.5 [21.9 - 113.0] μg/m^3
NO_2	20.26 + 4.11 × 10^{-5} × natural area, 500 m + 0.14 × length major road, 25 m + 3.60 × 10^{-5} × commercial-residential or industrial-residential mixing area, 500 m - 8.87 × 10^{-4} × urban green area, 100 m	0.74	0.63	6.36 μg/m^3	40	48.9 ± 12.2 [16.7 - 75.8] μg/m^3

The R^2 validation refers to the leave-one-out cross validation.

Table S2. Spearman correlation coefficients for air pollutants and the total lengths of major roads in 25, 50, 100, and 500 buffer zones.

	PM_{10}	$PM_{2.5-10}$	$PM_{2.5}$	$PM_{2.5}$ absorbance	NO_x	NO_2	MRL_25	MRL_50	MRL_100	MRL_500
PM_{10}	1.00	0.67	0.40	0.62	0.53	0.53	0.35	0.46	0.39	0.48
$PM_{2.5-10}$	--	1.00	-0.06	0.43	0.34	0.37	0.09	0.15	0.32	0.60
$PM_{2.5}$	--	--	1.00	0.25	0.34	0.21	0.28	0.39	0.28	0.05
$PM_{2.5}$ absorbance	--	--	--	1.00	0.50	0.47	0.50	0.40	0.35	0.40
NO_x	--	--	--	--	1.00	0.82	0.61	0.63	0.56	0.60
NO_2	--	--	--	--	--	1.00	0.57	0.47	0.41	0.53
MRL_25	--	--	--	--	--	--	1.00	0.80	0.50	0.13
MRL_50	--	--	--	--	--	--	--	1.00	0.72	0.18
MRL_100	--	--	--	--	--	--	--	--	1.00	0.38
MRL_500	--	--	--	--	--	--	--	--	--	1.00

MRL_25, the lengths of all major roads in a 25 m buffer; MRL_50, the lengths of all major roads in a 50 m buffer; MRL_100, the lengths of all major roads in a 100 m buffer; MRL_500, the lengths of all major roads in a 500 m buffer.

Table S3. Associations of systolic and diastolic blood pressures with an interquartile range increment of particulate matter and nitrogen oxides.

Exposures	IQR	Models	Systolic BP mm Hg (95% CI)	Diastolic BP mm Hg (95% CI)
PM_{10}	5.3 μg/m^3	Crude	0.11 (-0.16, 0.39)	0.39 (0.24, 0.55)
		Main	0.15 (-0.08, 0.38)	0.38 (0.23, 0.53)
		Extended	0.21 (-0.04, 0.45)	0.40 (0.24, 0.55)
$PM_{2.5-10}$	5.2 μg/m^3	Crude	-0.11 (-0.42, 0.19)	0.51 (0.34, 0.69)
		Main	-0.03 (-0.29, 0.22)	0.47 (0.31, 0.64)
		Extended	-0.02 (-0.27, 0.24)	0.47 (0.31, 0.64)
$PM_{2.5}$	4.0 μg/m^3	Crude	-0.01 (-0.23, 0.20)	-0.07 (-0.20, 0.05)
		Main	0.07 (-0.11, 0.25)	-0.02 (-0.14, 0.09)
		Extended	0.09 (-0.09, 0.28)	-0.04 (-0.16, 0.08)
$PM_{2.5}$ absorbance	0.4 ($10^{-5}m^{-1}$)	Crude	-0.03 (-0.30, 0.24)	0.30 (0.14, 0.45)
		Main	0.11 (-0.11, 0.34)	0.28 (0.14, 0.43)
		Extended	0.16 (-0.08, 0.40)	0.29 (0.13, 0.44)
NO_x	17.5 μg/m^3	Crude	-0.14 (-0.37, 0.10)	0.29 (0.15, 0.45)
		Main	-0.01 -0.21, 0.19)	0.30 (0.17, 0.42)
		Extended	0.04 (-0.19, 0.27)	0.35 (0.20, 0.49)
NO_2	6.7 μg/m^3	Crude	-0.03 (-0.27, 0.21)	0.44 (0.30, 0.58)
		Main	0.09 (-0.11, 0.29)	0.43 (0.30, 0.56)
		Extended	0.15 (-0.07, 0.37)	0.49 (0.35, 0.63)

The main models were calculated by generalized linear models, adjusted for sex, age, age mean-centered square, BMI, BMI mean-centered square, smoking status, alcohol consumption, education, hypertension, and diabetes. The extended models were further adjusted for traffic proximity in addition to covariates in the main models.

Table S4. Subgroup analyses for associations of systolic and diastolic blood pressures with increments of annual averages of particulate matter and nitrogen oxides stratified by physician-diagnosed hypertension.

Exposures (increment)	Systolic BP, mm Hg (95% CI)		Diastolic BP, mm Hg (95% CI)	
	Physician-diagnosed hypertension (n=12,702)	No physician-diagnosed hypertension (n=15,050)	Physician-diagnosed hypertension (n=12,702)	No physician-diagnosed hypertension (n=15,050)
PM_{10} (10 μg/m^3)	0.50 (-0.25, 1.25)	0.18 (-0.49, 0.85)	0.96 (0.57, 1.36)	0.30 (-0.05, 0.64)
$PM_{2.5-10}$ (5 μg/m^3)	0.03 (-0.39, 0.46)	-0.08 (-0.46, 0.30)	0.56 (0.33, 0.78)	0.29 (0.10, 0.49)
$PM_{2.5}$ (5 μg/m^3)	0.04 (-0.34, 0.42)	0.13 (-0.21, 0.48)	-0.02 (-0.22, 0.19)	-0.04 (-0.22, 0.14)
$PM_{2.5}$ absorbance ($10^{-5}m^{-1}$)	0.11 (-0.84, 1.06)	0.18 (-0.67, 1.04)	0.90 (0.40, 1.40)	0.34 (-0.10, 0.78)
NO_x (20 μg/m^3)	-0.09 (-0.48, 0.30)	0.08 (-0.27, 0.43)	0.39 (0.16, 0.62)	0.31 (0.10, 0.51)
NO_2 (10 μg/m^3)	0.06 (-0.45, 0.57)	0.25 (-0.21, 0.71)	0.76 (0.45, 1.06)	0.55 (0.28, 0.82)

Generalized linear models were used, adjusted for sex, age, age mean-centered square, BMI, BMI mean-centered square, smoking status, alcohol consumption, education, and diabetes.
